# Strategies for guided acoustic wave inspection using mobile robots

**DOI:** 10.1098/rspa.2021.0762

**Published:** 2022-03

**Authors:** Jie Zhang, Xudong Niu, Anthony J. Croxford, Bruce W. Drinkwater

**Affiliations:** Department of Mechanical Engineering, University of Bristol, Queens Building, University Walk, Bristol BS8 1TR, UK

**Keywords:** defect detection, defect localization, ultrasonic arrays, ultrasound image

## Abstract

Continuous non-destructive monitoring of large-scale structures is extremely challenging with traditional manual inspections. In this paper, we explore possible strategies that a collection of inspection robots could adopt to address this challenge. We envision the continuous inspection of a plate performed by multiple robots or a single robot that combines measurements from multiple locations. The robots use guided ultrasonic waves to interrogate a localized region for defects such as cracking or corrosion. In the detection stage, the receiver operating characteristic defines a detection zone in which a defect is thought to be present. In the localization stage, further measurements are made to locate the defect within this zone to a certain accuracy. We then address the question of what additional measurements are needed to achieve a given level of performance in the presence of uncertainty in robot locations? We explore this problem with Monte Carlo simulations that reveal the compromise between number of robots and performance in terms of defect location accuracy. In an experimental validation example on an aluminium plate, we show that six measurements arranged in a pentagon with a central measurement point leads to localization errors of similar magnitude to the uncertainty in sensor location.

## 1. Introduction

Structures such as buildings, bridges, tunnels, pipes and storage tanks must be routinely inspected to assess their structural integrity and reduce the probability of catastrophic failure [1]. However, these structures are typically large and access is often limited, both of which make these inspection tasks challenging. Many of these structures have geometries suitable for inspection using guided acoustic waves that are advantageous as they offer a reasonable compromise between sensitivity to damage and propagation range. For example, plates, rods and pipes have been inspected with guided acoustic waves in both permanently installed and deployable sensor systems. The longest range detection results (greater than 10 m) have been achieved at relatively low frequencies on structures that support one-dimensional wave propagation, e.g. pipelines [2–6] and rails [7,8]. In these applications, the transducers, which are attached externally, are either loaded against the surface or bonded with adhesive to affect a permanent installation. In all cases, a key requirement is to excite the desired single wave mode in a non-dispersive region [9,10]. For the permanently installed systems, further sensitivity can be obtained by subtracting baseline signals from the measured signals, where the baseline was acquired early in the life of the structure when it was in pristine condition. This is still an active area of research and the signals require compensation for benign changes relative to the baseline signals, such as temperature [11–13] and velocity [14–17].

If there are enough sensors around an area of interest, guided wave tomography [18–21] can be used for defect localization and characterization. However, this approach typically requires that the Nyquist–Shannon sampling theorem is satisfied, leading to the need for a large number of accurately placed sensors. Furthermore, in most incarnations, the signals from every possible transmitting and receiving sensor combination must be captured. For example, for a circular region of interest with a radius of 10 wavelengths, 125 sensors are required requiring the capture of 15 625 signals. This leads to accurate results, but also to high inspection costs. Hence, such an approach is suitable for monitoring certain critical regions and not entire structures.

A number of robotic systems now exist for automated inspection of large areas. The most well-known examples are the pipeline inspection gauges (PIGs). PIGs are inserted into a pipe and either pulled along using a tether or forced through under pressure. They have a number of sensors around their circumference and typically perform standard angled-beam ultrasonics or eddy-current inspection [22,23]. PIGs can provide 100% inspection coverage of large areas, but because of their high cost, are currently limited to high value assets, such as oil and gas pipelines. In addition, system disruption during insertion and removal is inevitable and these systems struggle to cope with varying cross sections or network complexity. Hence, PIGs are suitable for certain specific inspection scenarios, but are not generally applicable.

Remote-controlled mobile robots are also used for non-destructive inspections, particularly when access is difficult [24–27]. In some inspection scenarios, such as for simple geometries, these robots can be operated autonomously [28]. The cost of such robots has reduced dramatically over recent years leading to the possibility of deploying many small robots for an inspection [29]. For example, robots can carry an ultrasonic sensor to inspect storage tanks [30]; a robot with an ultrasonic sensor can inspect a floating production storage and offloading ship [25]; a robot with a mounted guided wave sensor can intelligently map a structure’s geometry and highlight the areas of significant wall loss [31,32]. Taking advantage of their ability to change their shape and functionality dynamically and reconfigure their communication connectivity, reconfigurable swarm robots [33] carrying different sensors have been recently used for safety monitoring of infrastructure such as nuclear waste storage drums [34], underground structures [35] and domes [36].

This paper takes the existence of small inspection robots as its starting point, and explores how they can be used for generic monitoring of a structure. This requires inspection strategies, methodologies and assessment procedures that can be integrated with the mobile robots for accurate defect detection and localization that is low cost and efficient. We investigate this problem by considering a network of robots, each with a single omnidirectional guided acoustic wave transducer. This configuration is considered as it is arguably the simplest, with good potential for integration in a low cost platform. The paper addresses only the inspection aspects of this interdisciplinary challenge, and we make the assumption that the inspection data and robot location data can be obtained and analysed. Even with these assumptions, this is an unbounded question with many different answers. Hence, our approach is to explore the simplest possible strategies first and consider what possible benefits further complexity might add. Given that robot location will always be subject to uncertainty, this aspect is considered from the start. We then aim to answer the question of how many robots are required to achieve a given level of inspection performance. Bringing these two aspects together we hypothesize that an inspection might be achievable with either a small number of accurately located robots or a larger number of robots whose locations are less well known. Relative to a fixed network, such mobile networks most obviously introduce uncertainty in sensor location, reducing their performance. However, they also have the advantage relative to a fixed network of reconfigurability. This second aspect means that the measurement density can be reconfigured when needed to perform specific tasks, such as localization.

Given the aim of the paper is to explore the simplest possible approach to mobile robotic inspection it is important to define what is meant by this. Here we consider simplicity to mean minimizing both the number of measurements and the associated data transfer. Doing this lowers both the complexity of the communication required and the energy requirements of the robot swarm. Hence we explore a two-stage approach in which the detection and localization stages are separated. Using this approach, the network is as sparse as it can be for detection, and is then reconfigured locally when additional measurements are needed, for example, to achieve some specified defect localization accuracy. Further, the post-detection spatial density of measurement points can be locally increased to achieve any desired performance.

This paper is organized as follows. Section 2 describes the inspection challenge and the core approaches to defect detection and localization. Section 3 describes an experimental realization that allows the Monte Carlo simulation of the inspection to be validated. In §§4 and 5 the detection and localization challenges are explored in further detail. In particular, the effect of the number of sensors and sensor location uncertainty on defect localization performance is assessed. In §6 the main results are discussed, as are a number of potential improvements to the detection and localization schemes. Finally, §7 summarizes the findings and draws conclusions.

## 2. Methodologies

### (a) Statement of problem

We assume a network of independent robots, each carrying sensors capable of both sending and receiving guided acoustic waves. We make no assumptions about the specific sensor performance in terms of signal-to-noise ratio (SNR) and this is left as a variable for exploration. The robots move through an inspection area where the first requirement is defect detection. In practice this would necessitate the specification of a critical defect type/size, around which the detection would be based. Once a defect is detected, a second requirement is to locate it to some specified accuracy. An additional step of defect characterization is possible, but is not explored in this paper. In the simplest possible detection scheme, each robot acts independently, with the network of robots providing the required inspection area coverage. This configuration has the major advantages of minimizing communication between robots, requires no synchronization and raises the possibility of on-board processing to lower data transfer costs. Note that in the subsequent analysis we only consider the presence of a single defect.

**Figure 1.**
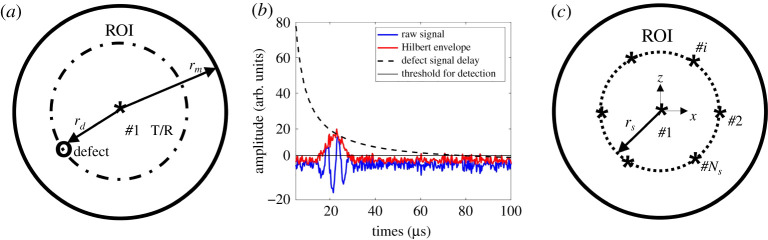
Schematic diagram of the acoustic guided wave inspection scheme. (*a*) The robot uses a transmitter/receiver in pulse-echo mode to monitor reflections, (*b*) the received data consist of a time trace from each robot location with one shown here. These raw data are enveloped to remove the phase information prior to processing, and (*c*) further inspection robots or locations can be used to obtain additional information about the defect, e.g. its location. (Online version in colour.)

Figure 1*a* shows a single sensor receiving a reflection from a defect at distance $r_{d}$. Defect detection is made when the amplitude of the time domain signal exceeds a defined threshold as shown in figure 1*b*. As the emitter is assumed to be omnidirectional, the guided waves propagate out from the sensor as a circular wavefront and each sensor location defines a region of inspection (ROI), which is a circle of radius $r_{m}$. Detection theory is used to specify $r_{m}$ based on a user requirement for a given probability of detection (POD) and probability of false alarm (PFA). To obtain 100% inspection coverage, the robots must move over the structure such that all points fall within the ROI of a robot at some point. This can be achieved with either a single robot scanning the area or a swarm working collaboratively.

After this first detection stage, the defect location is known, with some defined probability, to be within the ROI. To locate the defect with more precision, signals from other locations are required as shown in figure 1*c*. Here, sensor #1 has made the detection and defines the detection ROI. Further sensor locations are also shown within a second circle of radius, $r_{s}$. Hence, we envision the detection triggering a follow-up action that implements localization. This localization step requires data from a number of robot locations to be combined, for example, by the formation of an image. As with the detection stage, this could be achieved either by a single robot moving to a number of different locations, or by a number of robots coming together to achieve the same task.

### (b) Defect detection

The detection performance of a simple inspection system from a single sensor can be determined and assessed using the receiver operating characteristic (ROC) curve [37,38]. To form the ROC curve, we must know the probability distributions of the noise and defect amplitude. This then allows a user to balance probability of detection against probability of false alarms. The most appropriate operating point depends on the application, which means balancing the cost of missing a defect against the cost of reporting false alarms, which require follow-up actions, such as repair. In non-destructive testing applications, it is common to specify the POD and note that the lower the PFA at this POD, the better the detection performance.

The detection problem here is a binary problem in that a critical defect is either present or absent in the structure. The received signals are pulse-echo time domain traces, an example of which is shown in figure 1*b*. Each time trace corresponds to an instance in which a guided acoustic wave has been emitted and received by the robot-mounted sensor. Here, an amplitude threshold is set and a signal above this is categorized as a detection. Hence, there are four possible outcomes of a given measurement [37,38]: True positive (TP)—defect physically present and indication detected; False negative (FN)—defect physically present but no indication detected; True negative (TN)—defect physically absent and no indication detected; False positive (FP)—defect physically absent but indication detected.

In order to characterize the detection performance and select the most appropriate detection threshold, two quantities are defined: POD and PFA. POD, also known as the true positive rate (TPR), is the fraction of signals from physical defects that yield detectable indications,

2.1 $$\text{POD = TPR = }\frac{\text{TP}}{\text{TP + FN}}.$$

PFA, also known as the false call rate (FCR) or false positive rate (FPR), is the fraction of defect-free signals that will be wrongly flagged as containing defect indications

2.2 $$\text{PFA = FCR = FPR = }\frac{\text{FP}}{\text{TN + FP}}.$$

The relationship between POD and PFA as a function of threshold can be plotted on an ROC curve [37,38]. In the case of perfect detection performance, a threshold value can be found that enables a POD of unity and a PFA of zero to be achieved. However, in general, a compromise must be made between POD and PFA that depends on the requirements of the application.

In this case, we assume Gaussian white noise is present [39] in the received data due to thermal fluctuations, although the theory could be adapted for other noise distributions if required. Note that the Hilbert envelope (i.e. absolute value of Hilbert transform of signals) is applied to all time domain data as it acts to smooth signal phase variation in the raw time domain and thereby reduces the effects from possible phase error perturbed by noise and sensor mislocation [40]. Processing with a Hilbert envelope leads to a Rayleigh distribution of noise amplitudes, $a_{n}$ [41], with a probability density function (PDF),

2.3 $$P_{n}(a_{n})=\frac{a_{n}}{\sigma_{n}^{2}}\;e^{-\frac{1}{2}{\left(a_{n}/\sigma_{n}\right)}^{2}},$$

where $\sigma_{n}$ is the RMS of the noise amplitudes prior to enveloping. Its cumulative distribution function (CDF) is

2.4 $$C_{n}(a_{n})=1-e^{-\frac{1}{2}{\left(a_{n}/\sigma_{n}\right)}^{2}}.$$

Then, for an amplitude threshold of $a_{t}$,

2.5 $$\text{PFA}=1-C_{n}(a_{t})=e^{-\frac{1}{2}{\left(a_{t}/\sigma_{n}\right)}^{2}}.$$

The defect amplitudes, $a_{d}$, are assumed to be perturbed about their mean by the Guassian white noise, which, after processing with the Hilbert envelope, leads to a Rice distribution [42] with shape/scale factor υ,

2.6 $$P_{d}(a_{d})=\frac{a_{d}}{\sigma_{n}^{2}}e^{-((a_{d}^{2}+\upsilon^{2})/2\sigma_{n}^{2})}I_{0}\left(\frac{a_{d}\upsilon }{\sigma_{n}^{2}}\right),$$

where $I_{0}$ is the modified Bessel function of the first kind with order zero. The CDF is,

2.7 $$C_{d}(a_{d})=1-Q_{1}\left(\frac{\upsilon }{\sigma_{n}},\frac{a_{d}}{\sigma_{n}}\right),$$

where $Q_{1}$ is the marcum Q-function. Then, for the same amplitude threshold of $a_{t}$,

2.8 $$\text{POD}=1-C_{d}(a_{t})=Q_{1}\left(\frac{\upsilon }{\sigma_{n}},\frac{a_{t}}{\sigma_{n}}\right).$$

Note that υ can be calculated from

2.9 $$\upsilon =\text{argmin}\left(|\sigma_{n}\sqrt{\frac{\pi }{2}}\;e^{-\upsilon^{2}/4\sigma_{n}^{2}}\left[\left(1+\frac{\upsilon^{2}}{2\sigma_{n}^{2}}\right)I_{0}\left(\frac{\upsilon^{2}}{4\sigma_{n}^{2}}\right)+\frac{\upsilon^{2}}{2\sigma_{n}^{2}}I_{1}\left(\frac{\upsilon^{2}}{4\sigma_{n}^{2}}\right)\right]-{\bar{a}}_{d}|\right),$$

where ${\bar{a}}_{d}$ is the mean of $a_{d}$ from the same defect but different noise realizations.

For the guided acoustic wave signals considered here and shown in figure 1*b*, the amplitude from a given defect will decrease with distance from the sensor due to beam divergence and attenuation. Hence, the selection of the threshold, $a_{t}$, can be thought of as the selection of a distance at which the defect response amplitude falls below the threshold. This detection distance is $r_{m}$ shown in figure 1*a*. The higher the noise or the lower the detect signal, then the smaller $r_{m}$ is. The links between these key variables are explored in detail in the remainder of the paper.

### (c) Defect localization

Having detected the defect the next stage is to determine its location. The key question explored here is how many sensor sampling positions should be added to achieve a desired defect localization accuracy? In the scheme explored here, the information from the detection stage tells us only the size of the ROI, i.e. $r_{m}$. Given the circular geometry, we now introduce additional sensor sampling positions equi-spaced around a circle with a radius of $r_{s}$ as shown in figure 1*c*. Note, all configurations explored use the centrally located sensor that made the original detection as these data can be assumed to have been acquired.

To achieve defect localization across the ROI, we form an image of this region using the time domain signals acquired from the multiple sensor sampling positions. In this image, the amplitude of each image pixel is a weighted sum of contributions each time domain signal at the appropriate time of arrival. The pixel with highest amplitude in the image is selected as the measured defect location. This is implemented as a delay and sum imaging algorithm [40] where the intensity of the pixel at a location (x,z) can be written as

2.10 $$I(x,z)=\sum_{i=1}^{N_{s}}|H(s_{i}(t-\tau (x,z)))|,$$

where the subscript i is the index of sensor sampling position, $s_{i}(t)$ is the time domain signal acquired at the ith sensor sampling position, H denotes the Hilbert transform [43] and τ is the travelling time from a sensor to a pixel and back to the sensor. To investigate the effect of uncertainty in sensor location, the sensor locations can be perturbed relative to the desired locations with distances following a normal Gaussian statistical distribution with zero mean and a standard derivation of $\sigma_{p}$.

### (d) Wave scattering model

The performance of the proposed detection and localization schemes is explored using Monte Carlo simulations. This approach is needed to simulate many noise realizations as well as defect locations to predict the PDFs. A simple and fast wave scattering model is used to model ultrasonic signal as shown in figure 1*b* [40]. In this simulation, the guided waves propagate as circular wavefronts, are scattered by a defect with a known scattering response and then return to the sensor location where they are received. The total received signal is then the sum of this defect signal, $s_{d}(t)$, and some contaminating noise, $s_{n}(t)$.

2.11 $$s(t)=s_{d}(t)+s_{n}(t)=\frac{\beta \;e^{-2\alpha r_{d}}}{r_{d}}s_{0}\left(t-\frac{2r_{d}}{c}\right)+s_{n}(t),$$

where $r_{d}$ is the distance between the sensor and the defect, α is the material attenuation, $s_{0}(t)$ is the input signal, c is the wave speed and β is the defect backscattered amplitude coefficient that allows s(t) to match the experimentally received voltages [40]. β can be interpreted as the scattered amplitude when the defect is located at $r_{d}=1$ m in the absence of attenuation. This model assumes that the wave propagation is non-dispersive (a typical design aim, but we note that dispersion compensation could be applied if needed) and that the sensor is located in the far-field of the defect. We also performed a model in which we included the dispersion of the ${\text{S}}_{\text{0}}$ mode at our validation experiment operating point (see section 3(a)) and this revealed a difference in maximum amplitude of 3% after 265.6 mm of propagation, supporting the non-dispersive approximation. For simplicity, we also assume that the defect backscatters equally in all directions. We note that these assumptions could readily be removed in further investigations, however, the inspection scenario explored here is kept simple in order to reach generalizable conclusions.

## 3. Experimental realization

### (a) Experimental set-up

**Figure 2.**
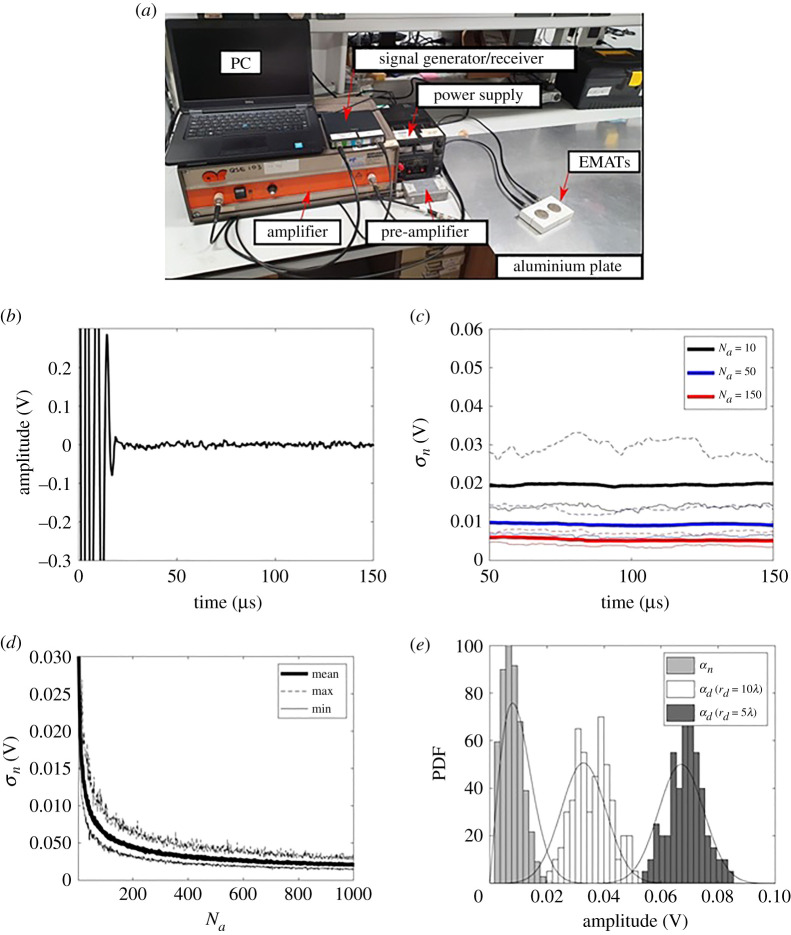
(*a*) A photo of the experimental set-up. Experimental measurements results from a 3 mm thick aluminium plate. (*b*) An example of noise signal obtained after 60 averages ($N_{a}=60$); (*c*) the noise RMS amplitudes ($\sigma_{n}$) within a 25 μs window as a function of $t_{w}$ and $N_{a}$; in each $N_{a}$ case, the bold solid line, the dashed solid line and the thin solid line represent the mean, maximum and minimum values, respectively; (*d*) $\sigma_{n}$ as a function of $N_{a}$, at $t_{w}=87.5\;\mu s$. (*e*) Comparison of the PDFs of noise and defect amplitude, $a_{n}$ and $a_{d}$, from the reference defect (8 mm diameter through-thickness hole) when $r_{d}=5\lambda$ and $r_{d}=10\lambda$. Note that these amplitudes are the absolute values of the Hilbert transform of the signals at $t_{w}=87.5$, 37.5 and 87.5 μs, respectively. (Online version in colour.)

We select a large aluminium plate of 3 mm thickness as an example test structure and use electromagnetic acoustic transducers (EMATs) for excitation and reception of guided waves as shown in figure 2*a*. Our intention is that this is simply an example to demonstrate and test the application of the described methodology. The methods, results and conclusions are applicable across a wide range of plate-like structures of different thicknesses and materials. The use of the experimental aluminium plate example means that (a) all parameters in the simulations are derived from experiment and (b) each step of the model is validated.

The example inspection uses the zeroth-order symmetric guided wave mode, ${\text{S}}_{\text{0}}$. This is excited at 200 kHz, which is below the first cut-off frequency in a region where it has minimal dispersion. We excite (and receive) this mode using a pair of co-located EMATs designed using a planar coil to apply predominantly in-plane displacement [44]. As such we excite ${\text{S}}_{\text{0}}$ with high levels of mode purity [44] and a measured group velocity of $5340\;{m\;s}^{-1}$, corresponding to a wavelength of λ=26.7 mm at the centre frequency. A further advantage of this mode is that it is also the fastest in this frequency-thickness range, making identification of the experimentally measured signals of interest straightforward.

It is noted that while this example is relatively thin compared with many engineering structures, EMAT sensors can also be applied to real-world structures, i.e. different materials and thicknesses and using different excitation schemes. For example, such sensors have been used to inspect a steel plate structure with thicknesses ranging from 5 to 10 mm [40]. The key point is to operate in a frequency range with low wave dispersion effects, typically keeping the frequency thickness product less than 1 MHz⋅mm for the used S_0_ mode. For example, this means that an operating frequency of 100 kHz could be used for inspecting 10 mm thick steel plates.

The transmitting EMAT was connected to the output channel of a signal amplifier 100A400 (manufactured by Amplifier Research, USA), the input channel of which is driven by an integrated digital oscilloscope and signal-generator device (Handyscope HS5, TiePie Engineering, The Netherlands); the receiving EMAT is connected to the input channel of a signal pre-amplifier matched to the EMAT sensors, the output channel of which is connected to an input channel of the same integrated digital oscilloscope. This digital oscilloscope is controlled by a PC and generates a five-cycle Hanning-windowed toneburst with a centre frequency of 200 kHz. The receiving pre-amplifier gain and the transmission gain on the transmitting EMAT were set to maximize the dynamic range of the measurement system.

The time delay added by the instrumentation and the length of the *dead zone* at the beginning of the received signals, due to the large amplitude cross-talk between the input and output channels, are important experimental parameters. These effects can be expected in any similar experimental set-up and we later show they have a particularly significant effect on the localization. These parameters were extracted from the signals reflected from a plate edge that was assumed to be a large planar perfect reflector. The measured system delay was 9.1 μs and the length of the dead zone was $t_{dz}\approx 30\;\mu s$, which is slightly longer than the pulse width of the input signal. Note that the system delay was removed for all experimental data by applying a back propagation step.

Figure 2*b* shows an example of a measured noise signal after 60 averages ($N_{a}=60$), where the dead zone can be seen in the first 30 μs. The noise root mean square (RMS) amplitude ($\sigma_{n}$) was extracted using a time window of the same width as the input pulse, i.e. 25 μs, at various starting times ($t_{w}$) as shown in figure 2*c*. As expected from random noise behaviour, $\sigma_{n}$ decreases with the increase of $N_{a}$ and is constant in time. As an example, for a specified window start time of $t_{w}=87.5\;\mu s$, figure 2*d* shows that $\sigma_{n}$ decreases with increased $N_{a}$. This provides a way to alter the noise level in the experiment so its effect can be studied. An example noise PDF obtained from 50 realizations is shown in figure 2*e* for $t_{w}=87.5\;\mu s$ and $N_{a}=60$. Also shown is the corresponding Rayleigh distribution (equation (2.3)), which can be seen to capture the shape of the distribution.

### (b) Calibration and validation of the simulation

**Figure 3.**
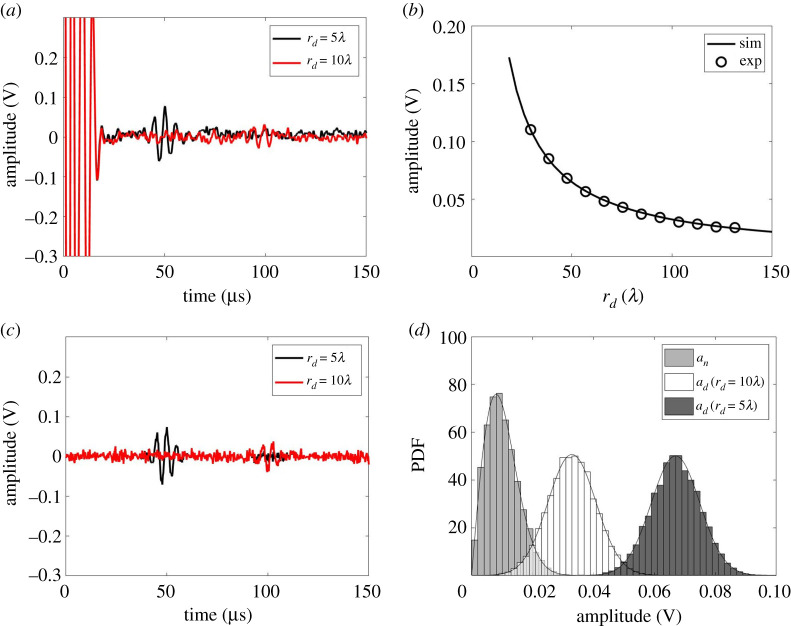
(*a*) Comparison of experimentally measured defect responses from the reference defect (8 mm diameter through-thickness circular hole) after 60 averages ($N_{a}=60$), when $r_{d}=5\lambda$ and 10λ; (*b*) the experimentally measured peak amplitude of the envelope of the signals reflected from the defect after 1000 averages at various $r_{d}$ (shown as circle symbols) and the best fit curve based on equation (2.11); (*c*) comparison of simulated defect responses obtained using equation (2.11) with $\beta =0.0083\;{V\;m}^{-1}$ and $\sigma_{n}=0.008\;V$, when $r_{d}=5\lambda$ and 10λ; (*d*) comparison of the PDFs of simulated signal amplitude from noise and the reference defect when $r_{d}=5\lambda$ and 10λ. The solid curves are evaluated using equation (2.3) for noise amplitudes and equation (2.6) for defect amplitudes. (Online version in colour.)

In both the experiment and the simulation, $s_{0}(t)$ is defined as a five-cycle Hanning-windowed toneburst with a centre frequency of 200 kHz. The random noise is modelled as $\sigma_{n}=0.008\;V$, which is equivalent to the noise level in figure 2*b*, i.e. 60 averages. Figure 3*b* shows the simulated and experimental defect response as a function of distance for an 8 mm (0.3λ) diameter through-thickness circular hole used here as a simple sub-wavelength reference defect. This curve was then used to extract the backscattered amplitude coefficient, β, to calibrate the amplitude of the simulated signals. Based on equation (2.11) $\beta_{\mathrm{ref}}=0.0083\;{V\;m}^{-1}$ using a least-square fit with an RMS error of 0.0007 V. Comparison between figure 3*a*,*c* shows excellent agreement between the calibrated simulations and the experiments.

The amplitude of the signal scattered from a small circular hole is proportional to the hole diameter [45]. Hence, for a circular hole with diameter, d, the corresponding backscattered amplitude coefficient, β, was obtained from the reference value ($\beta =0.0083\;{V\;m}^{-1}$) as $\beta =(d/d_{\mathrm{ref}})\beta_{\mathrm{ref}}$. Figure 3*d* shows the PDFs from the simulated signals which are also in good agreement with the experimental equivalents shown in figure 2*e*. Figure 3*d* also shows the analytical PDFs obtained using equation (2.3) for noise amplitude, $a_{n}$, and equation (2.6) for defect amplitude, $a_{d}$. They show excellent agreement with the Monte Carlo simulations. Figures 2 and 3 hence validate the assumption of Guassian white noise, the statistical characteristics of the Hilbert enveloped noise and defect amplitudes (equations (2.3) and (2.6)) and the wave scattering model used for the simulations (equation (2.11)).

## 4. Defect detection

**Figure 4.**
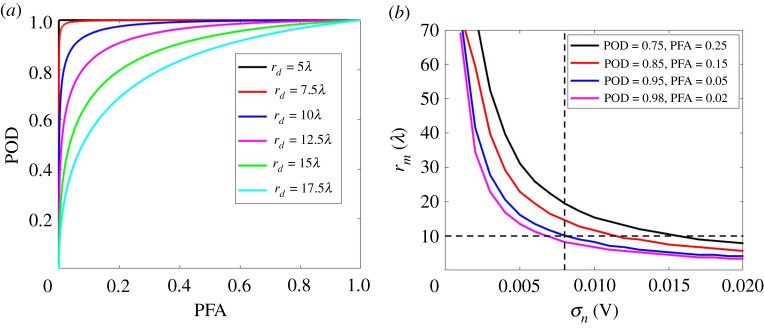
(*a*) Examples of the ROC curves for the reference defect with a noise level of $\sigma_{n}=0.008\;V$ at various $r_{d}$; (*b*) the maximum detection distance, $r_{m}$, as a function of $\sigma_{n}$, for the reference defect under various POD and PFA combinations. The results were obtained using equations (2.5) and (2.8). (Online version in colour.)

The wave scattering model described in §2d and validated in the previous section can be used to simulate signals from a wide range of plate-like structures. In any such scenario, the relative behaviour of the statistical characteristics of noise and defect amplitudes define the detection performance. As shown in figures 2*e* and 3*d* for the aluminium plate example, when $r_{d}=5\lambda$, the defect amplitude PDF is well separated from the noise amplitude PDF. In this case an amplitude threshold for defect detection can be set between the largest noise amplitude and smallest defect amplitude to detect the defect with no false calls. This leads to a perfect ROC curve as shown in the black curve of figure 4*a*. When $r_{d}\geq 7.5\lambda$, there is an overlap between the two PDFs (shown in figure 3*d*) leading to imperfect ROC curves. It can be seen from figure 4*a* that as expected the detection performance becomes worse as the defect location distance, $r_{d}$, increases. This happens because the defect amplitude decays with distance and beyond some distance the defect signals become subsumed by the noise.

The maximum detection distance of a sensor, $r_{m}$, is now used as a basis to design a statistically rigorous ROI. In our aluminium plate example, we can find the ROI for our reference defect (8 mm diameter hole) from the ROC curves. Figure 4*b* shows $r_{m}$ as a function of noise level, $\sigma_{n}$, for the reference defect under some particular POD and PFA combinations. For example, when $\sigma_{n}=0.008\;V$, POD=0.95 and PFA=0.05 then $r_{m}=10\lambda$. We note that this POD and PFA detection combination corresponds to an amplitude threshold of $a_{t}=0.02\;V$. It should be noted that $\sigma_{n}$ can be converted to SNR for the reference defect at $r_{m}$, hence this $a_{t}=0.02\;V$ corresponds to a SNR of 7.78 dB. Also shown in figure 4*b* are other possible combinations of POD and PFA and their effect on the ROI. The most suitable combination depends on the application needs and often the associated costs. In the remainder of the paper, we select POD=0.95 and PFA=0.05 leading to $r_{m}=10\lambda$ as an example for further exploration.

For the methodology described here, the sensor sampling path is governed by this detection stage. For the plate example, a possible inspection path could be a sequence of parallel lines with a separation distance of the maximum detection length minus the dead zone length. This path would ensure 100% coverage, if the sensor location was known perfectly. Sensor location uncertainty would then necessitate the use of more closely spaced scan lines to ensure that in the worst case scenario the specified $r_{m}$ is never exceeded. Although the robots act independently in the detection stage, if multiple robots are used then some cooperation clearly is required to achieve this type of scan sequence in an efficient manner with sufficient confidence. Discussion of optimal scan sequences is not the topic of this paper, but it is apparent from this discussion that the robots require some awareness of the location of the other robots in order to achieve the desired inspection coverage goals. In the next section, we show that this spatial information is also required for defect localization.

## 5. Defect localization

It is likely that manual inspection of the complete ROI will be cost prohibitive, and fail to take advantage of the robots present. Therefore, when a sensor registers a defect detection, the next stage in the inspection strategy is to localize the defect. Following the detection, we assume that possible defect positions are uniformly distributed in the ROI. To localize the defects, additional sensor sampling positions are required to acquire signals. After detection the angular location of the detect within the ROI is unknown and hence the sensor sampling positions are distributed around a circle to provide uniform angular detection coverage, as shown in figure 1*c*. It is known from the use of tomographic methods that the use of many sensor locations leads to high-quality images. However, here the challenge is to obtain an adequate image for localization, based on the use of the minimum number of sensor locations. Furthermore, if possible, the approach should be relatively insensitive to sensor location uncertainty. This is different from a permanently installed network in that the network here may be readily reconfigured for each of the tasks, detection, localization and characterization. We note that the detection stage can also reveal the defect range, and use of this additional information is explored in §6.

### (a) Image-based localization

**Figure 5.**
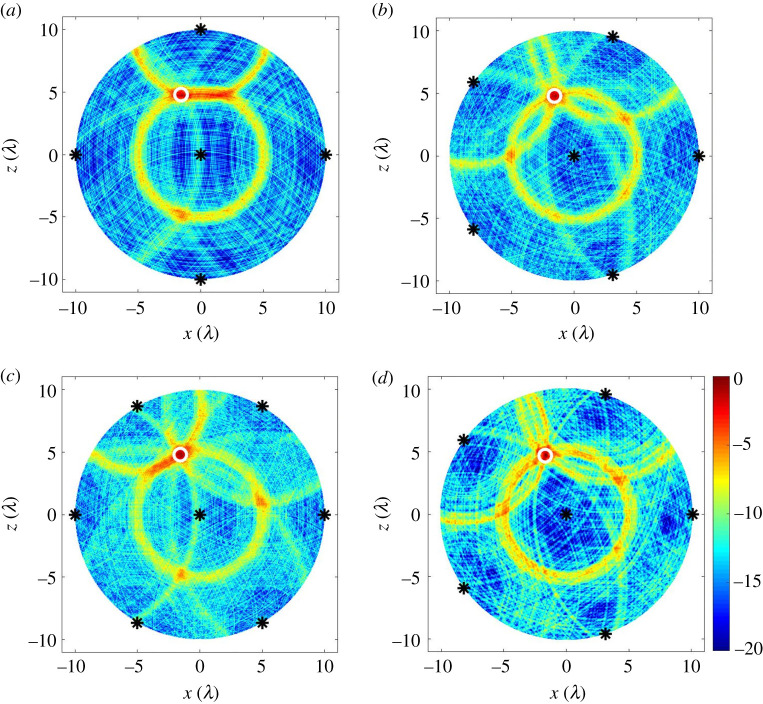
Guided wave images for the reference defect at (−1.57λ, 4.72λ) in: (*a*–*c*) simulated data when $N_{s}=5,6\;\mathrm{and}\;7$; (*d*) experimentally measured data when $N_{s}=6$. The simulated datasets were generated using equation (2.11) with $\beta =0.0083\;{V\;m}^{-1}$ and $\sigma_{n}=0.008\;V$. In each figure, The sensor sampling positions are labelled as black stars ($r_{s}=10\lambda$) while the defect location is a white circle. All images were obtained using equation (2.10), normalized to its own maximum amplitude and shown on a dB scale. (Online version in colour.)

Here we implement the use of the imaging algorithm defined in equation (2.10) for defect localization. In addition to the central detection sensor further sensors are added in a circle to ensure uniform angular coverage. As examples of specific realizations of noise and defect location, figure 5*a*–*c* show images using simulated datasets for five to seven sensor sampling positions. There is some observable improvement in the location performance in increasing from five to six sensor positions whereas increasing this further to seven sensor positions shows no apparent benefit. Comparing figure 5*b*,*d* reveals good agreement between images formed with simulated and experimentally measured data. The simulation reproduces the background noise levels and defect features providing further validation of the modelling accuracy.

It is important to note that the images in figure 5*a*–*c* are single examples drawn from a randomly varying set and overall conclusions must therefore not be made from these selected images. They do however provide a good qualitative picture of performance. Further discussion of how to select the most appropriate number of sensors based on stochastic simulations from multiple defect locations is made in §5c.

### (b) Image assessment metrics

The imaging performance can be assessed by the size of the image of a point-like target with amplitudes higher than a defined threshold (termed the point spread function), the SNR and the distance between the measured defect location and the actual one, termed the measured defect location error, $e_{l}$. As $e_{l}$ is the most direct measure of location performance, we now concentrate on this performance metric. The measured defect location is at the pixel with the maximum amplitude within the ROI, i.e. the circle defined by $r_{m}$ shown in figure 1*a*. Note that $r_{m}$ is fixed by the detection stage and the choice of POD and PFA combination.

**Figure 6.**
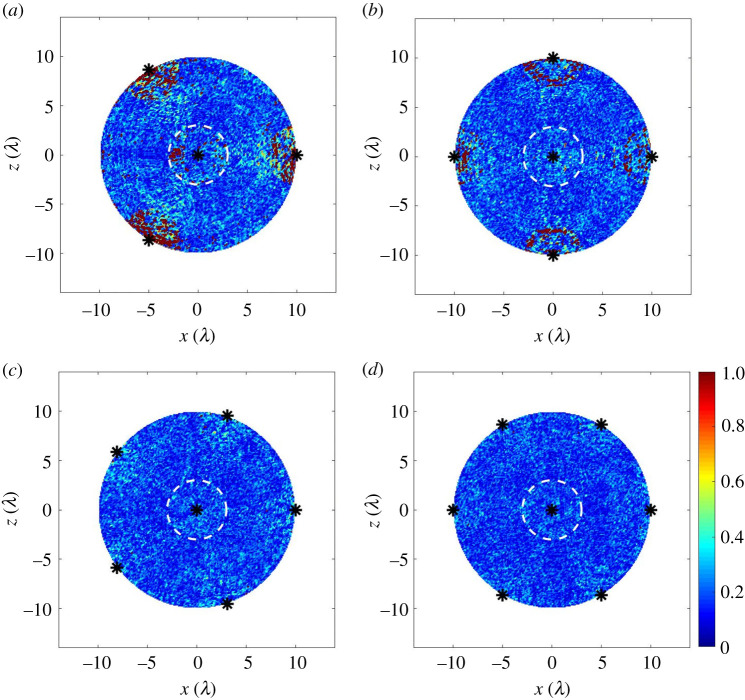
The measured defect location error, $e_{l}$, from the simulated datasets for all possible defect locations in the ROI ($r_{m}=10\lambda$), when $r_{s}=10\lambda$ and $N_{a}=$ (*a*–*d*) 4–7. In each figure, the dashed white circle has a radius of 3λ and indicates the dead zone of the sensor located at the centre. The separation distance between used defect pixel spacing in these images is 0.2λ in both x- and z-directions to produce a high-resolution image. This leads to 7845 simulated defect locations in the ROI. Only one noise realization was considered. The chosen maximum colour bar range is set to $e_{l}=\lambda$ to match the description in the text. (Online version in colour.)

Figure 6*a*–*d* show a localization performance simulation for one noise realization and all possible defect locations in the ROI ($r_{m}=10\lambda$). In particular, it compares the location error, $e_{l}$ when different numbers of sensor sampling positions are used. In this case, we aimed to keep location errors below λ although other values could be selected. As shown, when the number of sampling positions is less than six, there are a significant number of defect locations with $e_{l}$ greater than λ. These appear to be clustered around the sensor locations. This effect is caused by the sensor dead zone, meaning that one of the sensors does not contribute to the defect image. It is also shown that six sensor sampling positions are adequate to achieve $e_{l}$ less than λ across the ROI for this inspection example. Hence, this suggests the using more than six measurements is unnecessary for this location accuracy and would represent a poor use of robotic resources. Note that very similar results are obtained for other noise realizations.

### (c) Sensor sampling position

**Figure 7.**
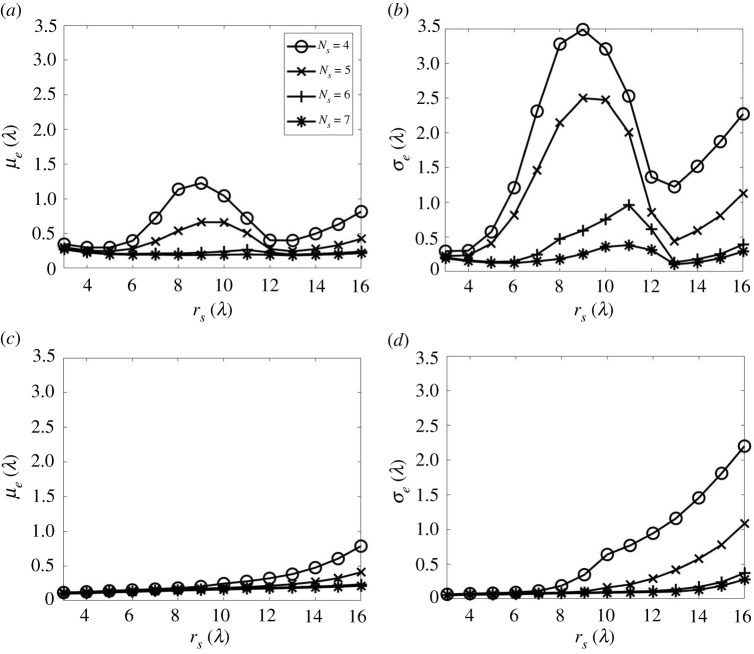
The statistical parameters of the measured defect location error, $e_{l}$, from the imaging simulation with: (*a*,*b*) including the dead zone of sensors and (*c*,*d*) excluding that. (*a*,*c*) are the mean of $e_{l}$ while (*b*,*d*) are its standard derivation. The simulated datasets are from six sensor sampling positions and 100 noise realizations. The other parameters considered in the data simulation are the same as those used in figure 6.

In the previous section, only one noise realization was considered and used to produce figure 6. Here the model is used to perform a Monte Carlo simulation, covering a large number (100) of noise realizations. In this way, figure 7 shows the mean ($\mu_{e}$) and standard derivation ($\sigma_{e}$) of the location error ($e_{l}$) across all defect locations within the specified ROI, calculated from 100 noise realizations. Figure 7*a*,*b* exclude data in the dead zone range, i.e. t<30 μs, as is typically needed to remove the cross-talk in any experiment. In figure 7*a*,*b*, a maximum value in the location error is observed at $r_{s}=8-10\lambda$, which is close to the limit of the ROI as $r_{m}=10\;\lambda$ in this scenario. The model also allows us to explore the effect of including the early-time data in the imaging, and thereby simulating the case where there is no cross-talk. The equivalent results of this simulation with no dead zone are shown in figure 7*c*,*d*. Hence, by comparing figure 7*a*,*b* with 7*c*,*d* it is apparent that the observed maximum in location error is caused by the removal of the dead zone data. This effect is particularly apparent for relatively low numbers of sensors, i.e. $N_{s}=4,5$ and is due to the removal of data that form the images in the vicinity of the sensors. As the number of sensor locations increases, this effect decreases, as the relative importance of data from any one of the sensors is reduced. As well as this dead zone effect, the other result seen in figure 7 is that, while there is a clear benefit in increasing the number of sensors from five to six, in terms of mean localization error, further increasing this to seven, only results in a further reduction of the mean localization error of 0.02λ. Hence, for this scenario, the use of six sensor locations provides a good compromise between location accuracy and cost in terms of measurement time or number of robots required (or both). It is also apparent from figure 7*a*,*b* that the sensors should be placed around circles of either $r_{s}=4-6\lambda$ or $r_{s}=13\lambda$ to minimize the influence of the sensor dead zones.

It should be noted that the number of sensors and their positions as optimized above are an example of the intrinsic compromise between defect location performance and the requirement to minimize the number of sensors/measurements. The aim of this section is purely to demonstrate how such a compromise may be arrived at. In practice, this decision would be end-user specific and depend on the required location accuracy, however the process outlined here would still be applicable.

### (d) Effect of sensor location uncertainty

**Figure 8.**
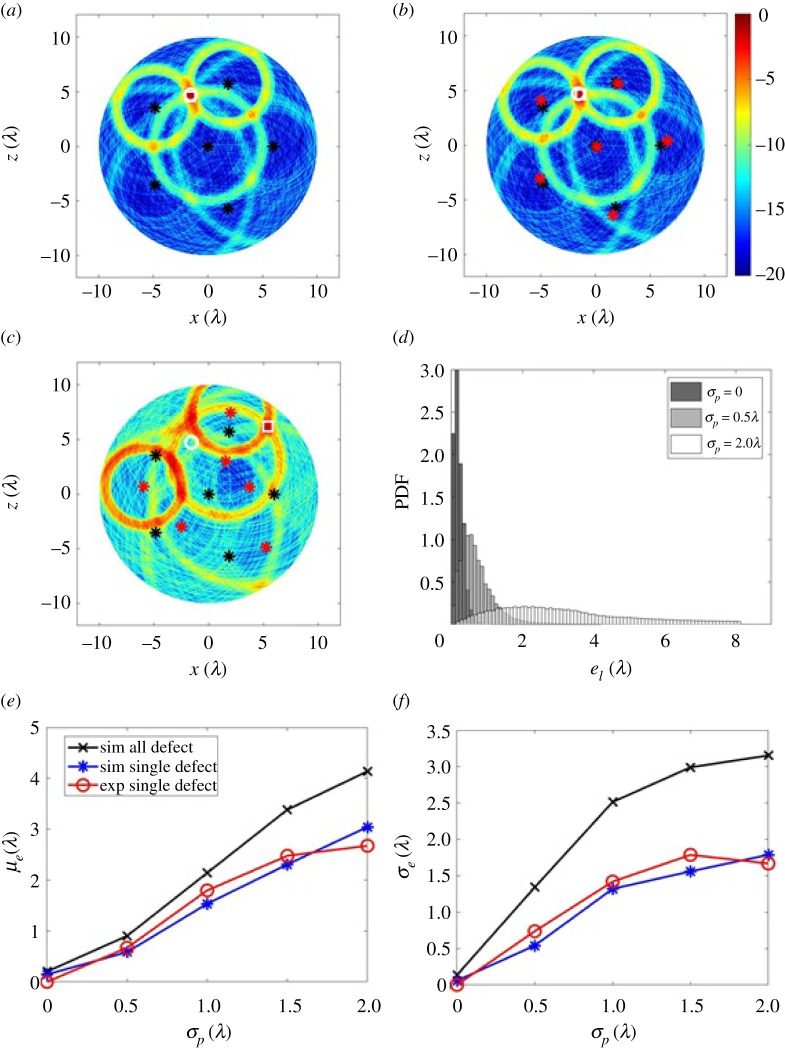
Investigating the effects of sensor misalignment, showing an ultrasound image from the reference defect at (−1.57λ, 4.72λ) with: (*a*) no sensor mislocation error; (*b*,*c*) a sensor mislocation error of $\sigma_{p}=0.5\lambda$ and 2λ. (*d*) Comparison of the PDFs when the sensor mislocation errors are $\sigma_{p}=0,0.5\lambda$ and 2λ. (*e*,*f*) $\mu_{e}$ and $\sigma_{e}$ as a function of $\sigma_{p}$. In (*a*–*c*), the sensor actual sampling positions are labelled as black stars while the mislocated ones (used in forming image) are red stars. The true defect location is marked as a white circle while the measured one is a white square. In (*e*,*f*), the line with cross symbols is from the simulated datasets for the reference defect at all 7845 locations in the ROI at the case of $N_{s}=6$ and $r_{s}=6\lambda$. For each defect location and each $\sigma_{p}$, 100 realizations of noise were considered. The line with star symbols is from the simulation data for the reference detect at (−1.57λ, 4.72λ); the line with circle symbols is from 15 experimental datasets after 60 averages. (Online version in colour.)

Uncertainty in sensor location is inevitable in practice and understanding its effect on the inspection performance is investigated here. The first objective is to quantify the effect of the sensor location uncertainty in terms of its impact on defect location error. This performance metric can then be used to determine the accuracy required by the robot navigation system. The second objective is to explore the hypothesis that the use of additional sensor locations acts to mitigate the resultant defect location errors. Both aspects are important as less prescriptive location accuracy would result in simpler cheaper robots with less complex communication requirements. Figure 8*b*,*c* show images using six sensors as the sensor locations are perturbed from their assumed locations. For comparison, figure 8*a* shows the image in the case where there is no sensor mislocation. The impression from these images is a sensor mislocation error of $\sigma_{p}=0.5\lambda$ has a relatively minor effect on defect location accuracy, whereas anything larger, e.g. $\sigma_{p}=2\lambda$, has a major impact on location accuracy. Figure 8*d*–*f* show the results of a Monte Carlo simulation of this scenario. Figure 8*d* shows the defect mislocation PDFs for various levels of sensor mislocation. This simulation was run over each defect location in the ROI, with 100 realizations of noise and 100 sensor mislocations, i.e. 10 000 simulations per defect location. From figure 8*d* we see that sensor mislocation leads to an increase in both the mean and standard derivation of the defect location error PDFs. This effect is also shown in figure 8*e*,*f* where the mean ($\mu_{e}$) and standard derivation ($\sigma_{e}$) of defect location errors are plotted as a function of the sensor mislocation error ($\sigma_{p}$). These plots show that the defect location error increases monotonically with sensor mislocation error. The mean defect location error can also be seen to be around twice the sensor mislocation error for this case, suggesting that sensor mislocation leads to defect location errors of the same order of magnitude.

**Figure 9.**
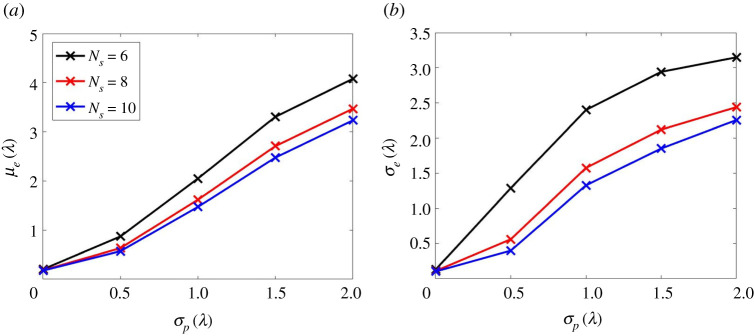
(*a*,*b*) $\mu_{e}$ and $\sigma_{e}$ as a function of $\sigma_{p}$ at various numbers of sensors. Note that the simulated datasets were from the reference defect at all 1257 locations with a separation distance of 0.5λ in the ROI ($r_{m}=10\lambda$), $\sigma_{n}=0.008\;V$, $r_{s}=6$, 100 realizations of sensor mislocation error and one realization of noise. Note that $\mu_{e}$ is not zero when $\sigma_{p}=0$. This is due to signals disturbed by noise. (Online version in colour.)

Figure 9*a*,*b* show the effect on the above errors when additional sensors are added. This suggests that, sensor mislocation errors can be reduced by using further sensors. However this coupling is weak. For instance, a 10-sensor network with a location accuracy of 1.5λ will have the same defect location error as a six-sensor network at λ location accuracy. This is important information that can be input to the design of the mobile robots and their navigation systems. For example, lower performance navigation will lower costs but take more time to acquire measurements due to the greater number of robots making more measurements. It is likely in practice that the design optimum will vary for each particular robotic swarm.

## 6. Discussion

In §§4 and 5, a two-stage methodology for guided acoustic wave defect detection and localization was proposed and implemented on a simple example structure. Monte Carlo simulations and experiments were then used to explore the influence of key variables, such as the number of sensors, on the detection and localization performance. As mentioned in §2 there are many potential methodologies that could be used in this type of scenario, and this paper explores one of the most simple approaches in detail. The variables discussed are all coupled and cannot be treated in isolation. For example, the quality of the sensing system and the time taken to record measurements will determine the level of noise, with a cheaper simpler system likely having more noise. The suggested detection range and sensor spacing for imaging will then follow from this first parameter. Similarly, the selection of location accuracy will propagate through all stages of monitoring with a group of robots.

In the first part of this discussion section, a number of alternatives/extensions to this simple approach are discussed and quantified. In the second part a number of additional implementation issues, such as the effect of more realistic defects and highly reflective geometric features, are discussed.

### (a) Improved detection based on images

**Figure 10.**
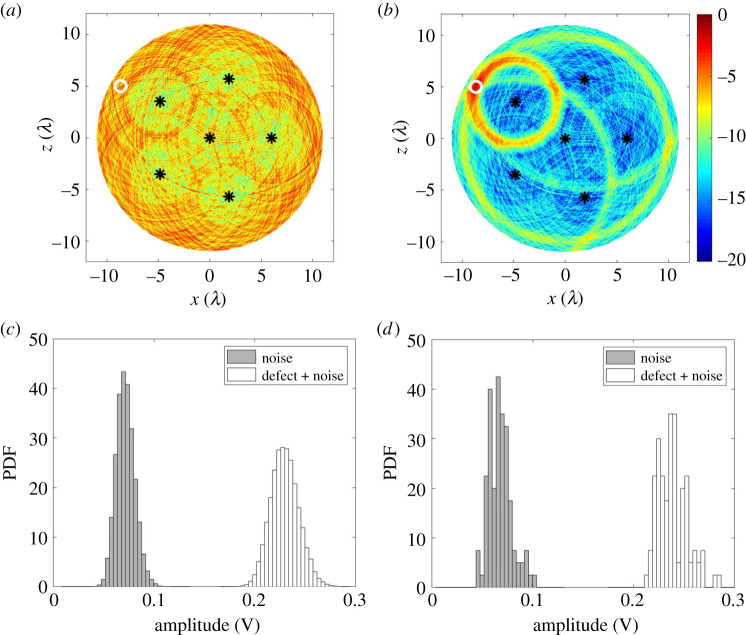
An example of simulated image from: (*a*) noise only and (*b*) the reference defect perturbed with noise. Comparison of the image amplitude statistical distribution at defect location (−8.7λ, 5.0λ) from noise and the reference defect at the case of (*c*) no sensor mislocation error and (*d*) $\sigma_{p}=2\lambda$. In the simulation, $\sigma_{n}=0.008$, $N_{s}=6$, 100 realizations of noise and 100 realizations of sensor mislocation errors were considered. (Online version in colour.)

Defect detection may be performed by fusing the data from multiple sensors [46,47]. The simplest approaches fuse sensor information at the data level. One readily accessible form of this is to form an ultrasound image, if the signals from multiple sensor sampling positions are available. Consider the detection of the reference defect (i.e. an 8 mm diameter hole) in the aluminium plate example using six sensors. A defect is placed at various locations around the $r_{d}=10\lambda$ circle. This circle marks the limit of the detection using a measurement from a single sensor based on POD=0.9 and PFC = 0.05. The noise and defect amplitude PDFs obtained from the images and shown in figure 10*c* are well separated and lead to a perfect ROC curve. This performance is also not significantly degraded due to moderate sensor position errors. As shown in figure 10*d* for $\sigma_{p}=2\lambda$ the noise and defect PDFs are still well separated, suggesting that image-based detection is relatively insensitive to sensor mislocation. This type of detection could be used across the entire inspection area, but is better suited to act as a follow-up to a single sensor detection. In the latter case, as multiple sensor locations are already needed for localization, this additional detection step would have only a minimal impact on system complexity. Hence, this second multi-sensor detection provides a route to reducing the effective false call rate of the system. Although this approach is an effective sensor fusion methodology it represents the simplest implementation. A more advanced approach built on feature fusion or decision fusion (for example, each sensor voting based on its decision) may be investigated in the future [48–51].

### (b) Use of sensor range information

**Figure 11.**
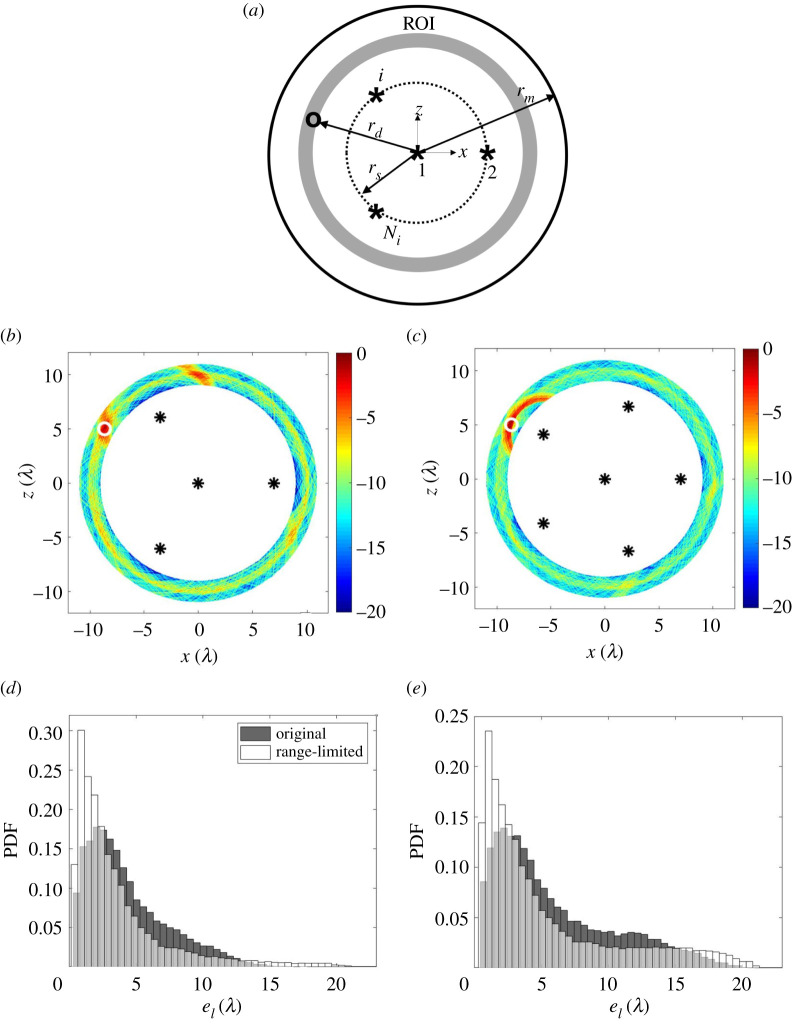
(*a*) Schematic diagram of sensor sampling positions with considering the extracted $r_{d}$ from the signal captured at the first sensor sampling position, termed the range-limited method. An example of the ultrasound image for the reference defect at (−8.7λ, 5.0λ), which corresponds to $r_{d}=10\lambda$, under a noise level of $\sigma_{n}=0.008\;V$, $r_{s}=7.0\lambda$ and $N_{s}=$ (*b*,*c*) 4 and 6. Comparison of the PDFs of the measured defect location error using the original and the range-limited methods for the case of (*d*) $\sigma_{p}=2\lambda$, $N_{s}=6$ and (*e*) $\sigma_{p}=2\lambda$, $N_{s}=4$. There is a grey ring in (*a*) which defines the region of interest for defect localization. The difference between $r_{d}$ and $r_{s}$ is the length of the dead zone of a sensor. For (*d*,*e*), 360 defect locations with equal angular space, one noise realization and 100 realizations of sensor mislocation error for each $\sigma_{p}$ were considered. (Online version in colour.)

Here the potential benefits of extracting range information from the initial detection stage are explored. From figure 1*b*, it is immediately apparent that the time of arrival of any detected defect signal can be measured. In addition, we would already need access to these data to enable imaging so there would be no additional data transfer costs. In this way, $r_{d}$ can be extracted from the signal captured at the first sensor sampling location. This range-limited method results in a significantly reduced ROI as shown by the grey ring in figure 11*a*. Note that here this ring is given a width the same as the toneburst to reduce the effects of uncertainty in the location of the detection sensor. It is then proposed to position other sensor locations in a circle as close as possible to the grey ring, such that the grey ring is not in the dead zones of the sensors, i.e. the dashed circle shown in figure 11*a*. The defect location can then be extracted from the peak amplitude location in the ring-shaped image, shown as figure 11*b*,*c*.

Figure 11*d* compares the PDFs using the original and this range-limited method for the reference defect in a circular region defined by $r_{d}=10\lambda$. For the case of a sensor mislocation error of $\sigma_{p}=2\lambda$, the mean error of the range-limited method is $\mu_{e}=0.16\lambda$, which is somewhat lower than with the original location method. Similar benefits are seen in figure 11*e* when four sensors are used. This result suggest that although including this range information can either improve defect location accuracy or reduce the required number of sensor locations, this effect is relatively small.

### (c) Information transfer requirements

This paper has focused on the use of co-located transmitter and receivers, i.e. pulse-echo mode. This has the advantage that the robots can act with a minimum level of information transfer between them, lowering the level of inter-robot communication required. In the detection stage, the robots require additional information about the location of the other robots for the purposes of path planning and inspection coverage. For the localization stage, if a single robot is used then the detection robot simply needs to navigate to the new locations, make the new measurements and then combine the received data to form the image. If multiple robots are available, then they need to know their location with respect to the detection robot to make the new measurements without collision. The robots then need to pass the received signals, along with their locations, to a single processing unit to form the image. This processing could be on the detecting robot or elsewhere. In all these cases, this information could be passed at any reasonable data transfer rate and so need not be expensive from a data transfer bandwidth perspective. It will ultimately be down to the operator to determine the ideal working condition, with one robot performing a greater degree of locomotion but less communications, or multiple robots moving on a simpler path but with a requirement for communication. This paper aims to give end users the tools to determine which represents the best use case.

When multiple robots are used, it is also possible for the guided acoustic waves that pass between them to be recorded. Such signals, termed pitch-catch in standard ultrasonic imaging, can provide additional imaging performance [40]. To use these pitch-catch signals to best effect the robots would need to share the same timescale to a high accuracy. This is because the receiving robot must know the time at which the sending robot emitted the guided wave. Potentially, this could be achieved with a wireless signal emitted simultaneously with the acoustic guided wave to mark zero time on the received signal, however this is currently challenging within the typical power budgets proposed for such robots but may be an area for future development. Nevertheless for completeness the impact of such additional data on performance is investigated below.

### (d) Using pitch-catch data

**Figure 12.**
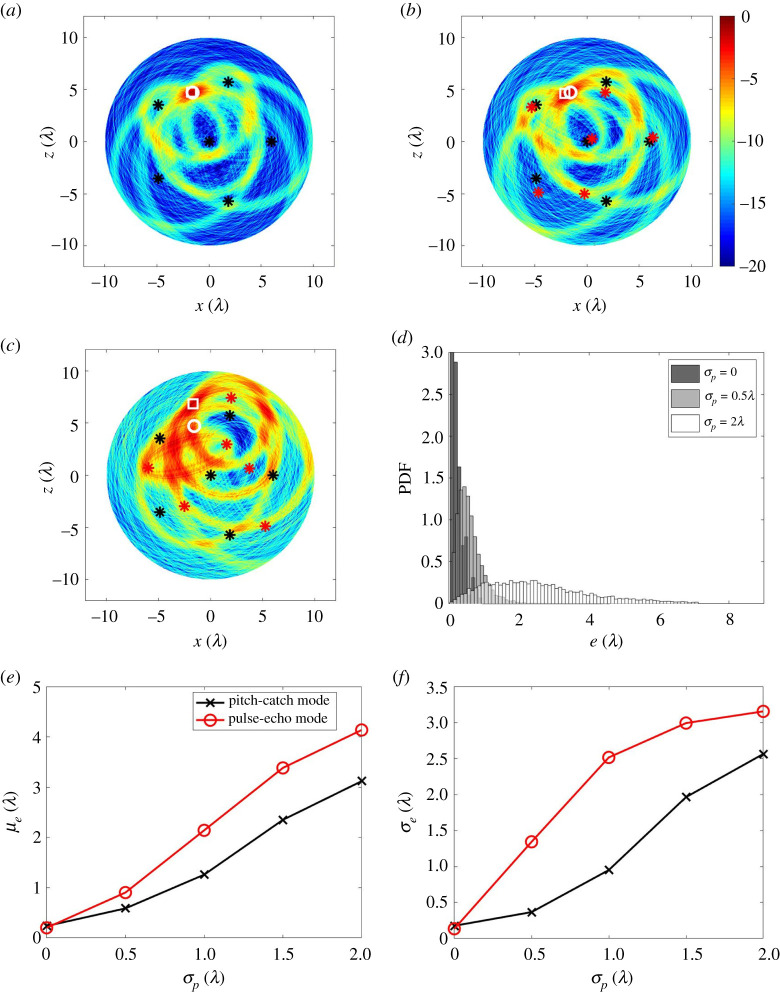
Investigating the effects of sensor misalignment under pitch-catch mode, showing an ultrasound image from the reference defect at (−1.57λ, 4.72λ) with: (*a*) no sensor mislocation error; (*b*–*c*) a sensor mislocation error of $\sigma_{p}=0.5\lambda$ and 2λ. (*d*) Comparison of the PDFs when the sensor mislocation errors are $\sigma_{p}=0,0.5\lambda$ and 2λ. (*e*–*f*) $\mu_{e}$ and $\sigma_{e}$ as a function of $\sigma_{p}$. In (*a*–*c*), the sensor actual sampling positions are labelled as black stars while the mislocated ones (used in forming image) are red stars. The true defect location is marked as a white circle while the measured one is a white square. In (*e*–*f*), the line with cross symbols is from the simulated datasets operating in pitch-catch for the reference defect at all 7845 locations in the ROI with $N_{s}=6$ and $r_{s}=6\lambda$. For each defect location and each $\sigma_{p}$, 100 realizations of noise were considered. For comparison, also shown as circles is the line reproduced from figure 8*e*,*f* for pulse-echo only localization. (Online version in colour.)

The paper has focused on the use of pulse-echo data, which is considered simpler in mobile robotics as it avoids the need for accurate time synchronization. However, here we explore briefly the impact of also including the pitch-catch data for localization. We assume that accurate time synchronization is possible and now collect all possible transmit-receive data from the robots. The additional data can simply be added to the imaging approach to localization described in equation (2.10). In this way, figure 12 shows a similar simulation to that carried out in the pulse-echo case (figures 8 and 9) except with the inclusion of pitch-catch data. Comparing figure 8 and figure 12, it is shown that, as might be expected, the additional pitch-catch data leads to improved localization, and lowering $\mu_{e}$ and $\sigma_{e}$. For example, assuming six robots, each positioned with an accuracy of a wavelength (or 26.7 mm in this case), the mean localization error, using pulse-echo only is $\mu_{e}=57.2\;\mathrm{mm}$ and when pitch-catch data is added is $\mu_{e}=33.6\;\mathrm{mm}$. Hence, for a given defect localization accuracy requirement the use of pitch-catch data would allow a reduction in the number of robot locations, or, more likely, it could be used to simply enhance the localization performance. Each of these would of course come at the expense of measurement and equipment complexity.

### (e) Comparison with rectilinear scanning approach

There are many alternative ways in which robots could be positioned and scanned to cover a structure. One simple alternative is to arrange the robot measurement locations as a rectilinear grid over the area of interest, similar to the widely used C-scan method. In this case, a key variable is the measurement pitch, i.e. the average separation of the robots/measurements. If this is similar to our $r_{m}$, then it is reasonable to conclude that the C-scan achieves similar detection levels to our proposed detection stage. It may be somewhat better at some points interrogated by several sensors. But this improvement will be small, as the sensors outside $r_{m}$ receive only noise (that is the definition of $r_{m}$). Such a set of measurement locations would also be very poor from a localization perspective, especially given the measurement location uncertainty. Hence, it is apparent that the measurement pitch in the C-scan approach would have to be smaller than that for our proposed detection stage. Indeed, to achieve similar levels of localization to the proposed two-stage method, the pitch of the C-scan would have to be similar to the spacing of the measurements we propose to use only for the second localization stage. It then seems reasonable to argue that this C-scan must therefore be less efficient as it requires a smaller measurement pitch over the whole structure and continuous flow of data between robots to form images (or implement some other localization algorithm).

### (f) Other implementation issues

In this article, a defect was assumed to backscatter equally in all directions, hence its scattering at the inspection frequency could be included in the simulation by a single parameter, β. The experiments used a small (diameter 0.3λ) through-hole to approximate this situation. This geometry could represent small regions of pitting, corrosion or erosion. This simple defect also allowed us to simplify the inspection scenario leading to ready interpretation of the results. However, real defects have arbitrary geometries and different scattering behaviours. Defects that are planar in shape, such as cracks, scatter in specific patterns. In particular, they exhibit strong backscattering when the wave is incident normal to their face and much weaker backscattering when the wave is incident on the crack-tip. This scattering also varies with crack size, becoming more strongly directional for larger cracks [52]. The main implication for the detection and localization methodologies is that the backscattering amplitudes will also depend on the angle between the crack normal and the sensor. One simple way of including this variable scattering in the statistical framework described here is to set the detection based on the worst case scenario, i.e. the crack normal perpendicular to sensor angle with the smallest backscatter amplitude. Hence, if this worse case orientation can be detected, it may be assumed that all other orientations can too. However, this worst case concept is overly conservative as each defect will be insonified from a number of directions in the detection stage as the robot moves over the structure. An extension of this idea is to use scan paths that guarantee each point is inspected from a range of angles, for example, by performing two rectilinear scans, the second rotated with respect to the first by some angle. This then ensures that the lowest backscatter angle does not set the detection limit. This is precisely what happens when multiple sensors are used to form an image as described in §5, i.e. each location in the ROI is inspected from different angles by multiple sensors. Hence, a move to multi-location detection will be more robust to the variable scattering from crack-like defects. Such an approach will ultimately lead to a trade-off between scan pitch and $r_{m}$ and would need to be studied in detail for angular-dependent defects.

In addition to detection and localization, using multi-sensor locations also opens up the future possibility of defect characterization. The approach would be that once located, the angular reflectively pattern of a defect could be measured. This would probably require further measurement locations to obtain full angular coverage [53]. We note here that similar concepts have been explored in tomography [19–21] and in array imaging [54,55]. In the array imaging case, the measured angular reflectivity signature is compared with a pre-computed database of scattering responses. The best fit is found and used to characterize the measured defect [52].

In the simulation, the plate has no edges and so signal can only originate from a defect. Also the defect positions are assumed to be uniformly distributed in the ROI. In the experimental example, a large plate was used to reproduce this effect. In many real-world structures such a large pipes, this assumption is often reasonable, however, a large number of real structures have edges and geometric features such as changes in thickness. Defects could have a higher possibility to appear in areas with high concentrated stress. Further work is required to explore how the proposed methodologies could be applied to these situations. Imaging, using multiple sensor locations, is a way of coping with the edges as they will appear in the images as spatially distinct features. If the robots had some knowledge of the location of the edges, then these regions could be excluded from the images. Alternatively, more sensor locations could be required near the features to obtain higher quality imaging, aiding classification of the detected signal as either a geometric feature, or a potential defect.

In the scenario described, while the robots are mobile, the measurement is static due to the requirement to capture a number of averages in each location to achieve an adequate SNR. This is thought to reflect the situation in a real scenario where low energy availability on the robot will necessitate low amplitude transmissions needing averaging. If it were possible to measure while moving, inspection speed could be increased as would the potential to inspect from many more locations. However, if the robot was measuring as it moves, then the currently used simple averaging approach would not be possible. Although some other averaging scheme could potentially be developed to overcome this, such a scheme would also have to cope with the positional uncertainty that goes hand-in-hand with mobile robotic systems.

It should also be noted, e.g. by considering figure 8, that due to the inherent location inaccuracies, the sensors are only approximately circularly arranged. The reality is that they will be randomly distributed on a circular seed. It is possible that other seed patterns may offer performance benefits and would benefit from further study. The assessment procedure and the process of analysis proposed in the paper can be used for exploring and optimizing the performance of such different sensor patterns.

In this study, we have only considered single defects in a region, however, if multiple defects are present the performance of the current approach is unclear. The importance of this multiple defect scenario will depend on the use-case. If multiple defects are a concern, an alternative way of using the current approach is as a screening tool. Once a defect is found and located, some follow-up action would occur, for example, a visual inspection or a repair. Critically, at this follow-up stage there is the possibility to implement techniques to identify the presence of multiple defects. This may be possible with the addition of more guided wave measurements, but it may also be better achieved with other modalities, such as visual inspection. This is an area that requires significant further study.

## 7. Conclusion

A methodology by which mobile robots can inspect large plate-like structures (as effectively seen in large pipe sections) has been described. In this paper, the process was divided into a detection stage and a defect localization stage. In each case, the measurement strategies are different, leading to different trade-offs and optima. In the detection stage, a robot is moved over the structure and detection is performed at every location. Once a feature is detected, this is followed up by further measurements in the vicinity of the detection. We have shown how to define the detectable range and thereby localize the defect within a region, based on the selection of a required POD and PFA. The localization stage was shown to improve significantly with up to six sensors, for an aluminium plate example. The localization stage was also shown to be robust to the sensor location errors, due for example to robot navigation errors. For the six-sensors scenario, the resulting defect location errors were around twice the sensor location errors.

While we explored a specific aluminium plate inspection example, the methods described are generally applicable to other related scenarios. The key requirement is a fast model of the inspection that allows Monte Carlo type simulations of the defect and noise PDFs. This approach then allows the impact of any detection or localization method decisions to be quickly quantified. For example, the approach could be used across other materials, plate thicknesses, noise levels or guided wave modes, allowing the full range of sensor performance parameters, defects sizes and types and operating modalities to be explored. For any of these scenarios, the method can be used to determine the minimum number of sensor sampling positions and achieve a pre-defined defect localization performance. The methodology can be integrated with robot navigation for efficient inspections.

A widely applicable detection and localization scheme has been presented and explored in detail. We have also described a number of enhancements to this scheme. In particular, if the data from multiple sensor locations are combined, then improved detection can be achieved. This could enable a reduction in the false calls and provide a route to coping with reflections from geometric features such as edges. The range information can also be extracted from the detection stage, and this provides a better initial localization. Our results suggest that this approach can be used to either reduce the number of sensors, or counter the effects of sensor location errors. Together these strategies demonstrate a workable approach to the use of autonomous (or semi-autonomous) mobile robots for the inspection of large structures that is just the beginning of exploration in the area.

## Data Availability

All data and codes are available from the Research Data Repository of the University of Bristol (http://dx.doi.org/10.5523/bris.3faz3idnijk9d2vlhj3vv40dxu).
